# Views on everyday life among adults with spina bifida: an exploration through photovoice

**DOI:** 10.1080/17482631.2020.1830702

**Published:** 2020-11-04

**Authors:** Hanna Gabrielsson, Claes Hultling, Agneta Cronqvist, Eric Asaba

**Affiliations:** a Faculty of Medicine and Health, School of Health Sciences, Örebro University, Örebro, Sweden; b Department of Health Care Sciences, Ersta Sköndal Bräcke University College, Stockholm, Sweden; c Spinalis, SCI Unit, RehabStation Stockholm, Stockholm, Sweden; d Spinal Foundation, Stockholm, Sweden; e Sophiahemmet College, Stockholm, Sweden; f Department of Neurobiology, Care Science and Society, Karolinska Institutet, Stockholm, Sweden; g Department of Neurobiology, Care Science and Society (NVS), Division of Occupational Therapy, Karolinska Institutet, Stockholm, Sweden; h Unit for Research, Education, and Development, Stockholms Sjukhem Foundation, Stockholm, Sweden; i Occupational Science and Occupational Therapy Research Group, Lunds University, Lund, Sweden

**Keywords:** Spina bifida, adults, photovoice, community-based participatory research

## Abstract

The aim of this study was to actively integrate expertise of persons living with spina bifida, to explore conditions embedded in their everyday life. This was important because young adults with spina bifida risk not being able to fully participate in the community on equal terms and in accordance with their own preferences. Photovoice, a community-based participatory research approach, was utilized to engage participants through dialogue and photography. An exhibition was created to share results with community and stakeholders. An overarching theme that characterized the experiences of the group was, “an adaptation for us, but it works for no one”. Findings are presented as: “*Accessibility—a never-ending project*,” *“Tensions of a normative view*,” and “*Power to influence.”* Findings integrated everyday life metaphors photographically depicted by broken elevators, unsafe transportation, closed doors and not experiencing real opportunities of involvement. Tensions in everyday life experienced by persons living with spina bifida can inform conditions relevant and necessary to support community participation, particulary among persons living with disability.

## Introduction

It has been reported that youths with spina bifida (SB) are at risk of not being able to fully participate in community on equal terms and in accordance with their own wishes (Kelly et al., 2011; Lindsay, 2014; Soares et al., 2008). For instance, it has been described as difficult for persons with SB to “make sure to get things done” and “make things happen,” without relevant support (Gabrielsson et al., 2015). Young adults with SB have reported a lack of motivation as a barrier, not only to community participation (Boudos & Mukherjee, 2008), but also to being physically active (Buffart et al., 2009). Moreover, a protective attitude by those around persons with SB have been described as a barrier to participation in physical activity among persons with SB (Bloemen et al., 2015), and differences in independence and in response from the environment have been shown to contribute to conflicting feelings about meeting others with SB (Gabrielsson et al., 2015).

Spina bifida is a congenetial and rare condition (10–20/100.000 newborn children/year) (Bodin et al., 2018). Adults living with SB often have physical and cognitive impairments to varying degrees, which can be one reason why healthcare research in the area of SB has primarily focused on impairments and disability in young age. Studies examining adult experiences of SB are scarce as well as studies integrating in research the voices of people living with spina bifida. Although it can be important to identify challenges experienced by persons with SB, in order to design improvements and solutions in everyday life for this group, it is also important to challenge the language of inability in favour of engaging those who live with SB in reframing the focus of what is important from an insider perspective. A language of marginalization begins early, and despite possible challenges, there is a need to integrate the experiences and voices of persons living with SB in the knowledge continuum that addresses SB in society. This is in line with the United Nations (UN) Convention on the Rights of Persons with Disabilities (CRPD) (United Nations, 2008). Furthermore, setting the scene of this study on the basis of the CRPD is relevant because in Sweden, this convention has informed disability policy and the national goal to increase participation in society for all people (The Swedish Agency for Participation, 2019).

In this paper, “participation” is used to mean more than just being involved in situations. It involves (a) active and meaningful engagement/being part of; (b) choice and control; (c) access and opportunity/enfranchisement; (d) personal and societal responsibilities; (e) having an impact and supporting others; and (f) social connection, and social inclusion and membership (Borell et al., 2006; Hammel et al., 2008; Hemmingsson & Jonsson, 2005). The concept of participation and actively involving people in research design to relocate the voice of persons with disability at the centre, rather than at the periphery has been raised as critical for decades (Knox et al., 2000). Photovoice in particular has been used in several studies where persons with intellectual and spine related disabilities or rare disease have collaborated in advancing the community practices as well as research and policy agendas (Jurkowski, 2008; Holmlund et al., 2018; Mälstam et al., 2018; Newman, 2010; Povee et al., 2014, & St. John et al., 2018). The aim of this study was to actively integrate expertise of persons living with spina bifida, to explore conditions embedded in their everyday life.

## Material & method

### Design

Participatory research methods were utilized to include and empower relevant stakeholders. This is relevant because although expert-driven research makes many important contributions, such as in many intervention studies, it can also perpetuate certain discrimination by excluding the voice of the persons about which the research is intended. Participatory research places participants in an active role in the research process, challenging traditional power dynamics, thereby opening up for alternative research processes. The project design is grounded in the idea that results need to be personally meaningful and relevant for the people in the study and for their surrounding community. Ethical approval was obtained from the Regional Ethics Board (Dnr: 2017/992-31/2).

### Participants

The Swedish patient organization for adults with SB, Spin-Off, were contacted and provided with information about the study. This information was also provided by email to the individual members of the organization, along with a request to participate. Those members interested in participation were contacted by telephone and given more information about the project, and received an invitation to an introductory meeting. A total of nine persons were interested in participating in the project, but four declined participation after receiving the information. The final group therefore consisted of five members, four men and one woman aged between 30 and 49 years. The number of the group members was in keeping with photovoice methods and allowed for active dialogue among all group members (Asaba et al., 2015; Minkler, 2005). Of the members, three lived alone, one with 24-h assistance, and two lived in serviced apartments. Two worked part-time with subsidy, two had sheltered work and one had early retirement. Four of the group’s members used a manual wheelchair and one walked. Four were Swedish-born: one person had moved to Sweden around the age of 4.

### Photovoice

Photovoice was utilized and is a community-based approach, consisting of participatory methodologies and methods, which is collaborative and action-oriented (Asaba et al., 2015; Minkler, 2005). Feminist theory, documentary photography, and empowerment theory are cornerstones in informing the research process including community partnering, data generation and analyses, sustainability, and communication of findings. Photovoice can be distinguished from other methods where photos or pictures are used, in that members in a photovoice group are active in revising and co-designing the questions posed, for example by actively make decisions about what and how to generate photos. Photovoice aims to engage people as experts on their own life situation through dialogue, narratively and using photography. The approach has a clear commitment to involve people who, in different ways, are described as living with some degree of or risk for marginalization (Plunkett et al., 2013). Photovoice is relevant in that it facilitates: (a) actively engaging people in reflection and documentation of strengths and concerns in their communities, (b) promotes and facilitates critical dialogue, and (c) reaches beyond the academic domain to open up a space for dialogue to influence policy makers (Wang & Burris, 1997; Wang & Redwood, 2001).

### Data generation

Photos in the photovoice method served as a starting point for the discussion about what was of interest in daily life. Each week the group formulated a theme, based on which the members took pictures. Four of the group members used their phone for photography; one received a compact camera for the project. One group member chose to also bring photos taken in the past to the sessions.

**Figure 1.**
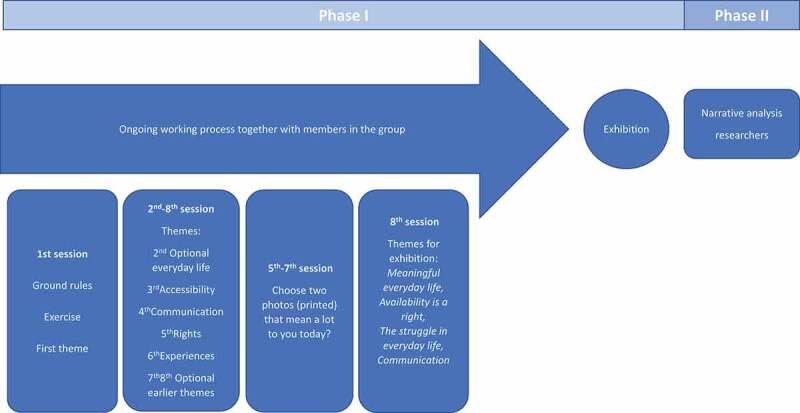
Flowchart of the research process

The group met once a week for 8 weeks, for sessions lasting 2 hours. At the introductory session, the aim of the study was presented, and the researchers explained the photovoice procedure. Ground rules were agreed on, such as aiming on be on time, and to listen to each other and agreeing that what was said in the room was to stay in the room. Informed consent was reconfirmed. Individual arrangements were made if the members wished to be reminded during the week. Also, an exercise involving selecting and talking from an already existing photo was carried out together. Further, the question of ethical considerations when taking and sharing photos about others was raised. The group agreed to bring three photoseach per week, to work with during the sessions. For each session, a theme was formulated by the members, as presented in Figure 1. The discussions of the themes continued across the sessions; for example, accessibility was discussed during many of the sessions, not only during the session “accessibility”.

Two facilitators guided the group. The role of the facilitators was to ensure that the process of photovoice methodology was followed, organize the sessions (logistics, technicalities, keeping track of time) and support the group in working together (Asaba et al., 2015; Wang & Burris, 1997). In this study, a third facilitator was engaged especially in the stage of preparing an exhibition. All sessions were held in Swedish. The translation of the quotations from the transcripts into English were done by a professional translator.

### Data analysis

The analysis consisted of two phases, as seen in Figure 1. Phase I involved the photovoice process, from group meetings and visual analyses to the final exhibition. Phase II consisted of an narrative analysis conducted by the researchers.

#### Phase I

The members decided each theme from week to week, and accordingly brought photos displaying that theme to each session. When sharing the photos, the members in the group spontaneously discussed the experience and possible meanings. To support the process, the SHOWeD method was utilized as a guide (Shaffer, 1985), using the questions: What do you See here? What is really Happening here? How does this relate to Our lives? Why does this concern, situation or strength exist? How can we become Empowered through our new understanding? And, what can we Do? The last question, What can we do? eventually led to a decision to show some of the photos in an exhibition with the ambition to share the findings with others and hopefully reach stakeholders to show what everyday life with SB can consist of. This is congruent with the third goal of photovoice (Wang & Burris, 1997).

In session 4–7, the whole set of photos for each theme was displayed, and the members choose two photos each that were most relevant to them that day (see Figure 1). These photos were posted on a wall and grouped into exhibition themes. The photos were grouped under the following headlines: *Meaningful everyday life; Availability and accessibility is a right; The struggle in everyday life*; and *Communication*. To reason together, out loud around an image or several images, was a way of co-creating meaning for the chosen photos and at the same time contributing to a visual analysis by categorizing and assigning meaning to constellations of photos. A text accompanying the photos was created by the group and stemmed mainly from the sessions. Each session was recorded and transcribed verbatim.

The themes for the exhibition were worked with during the last two sessions. Careful considerations for the members’ integrity were taken when prioritizing photographs, and ethical considerations was given to the right to anonymity and privacy of other persons who were visible in any of the photographs. Since the group was united in its decision to have an exhibition the members all agreed to break their anonymity and signed another consent regarding this.

#### Phase II

The narrative analysis (Polkinghorne, 1995) continued to build on Phase I, and drew on transcripts, photos and fieldnotes from the eight sessions. The initial reading of transcripts focused on identification of significant events combing transcripts for situations that were distinctive, representative or surprising. A surprise could be a finding that was contradictory to previous material, which either came from the intra- or inter-participant research data, or was based on variation from the literature. As significant events were identified from the transcripts, the first and last author began to reconstruct a narrative storyline around the event, discussion, and pictures that had been shared during a session. By reconnecting fragments from the experiences shared and the stories told, common themes could be identified that had a focus on actions and happenings grounded in the data (Polkinghorne, 1995). The themes include representative stories from photovoice sessions that were shared in tandem with photos, fieldnotes from the first author, and photos from the sessions. The themes were discussed among the researchers to reach richness and to check for representiveness against raw data. What is unique in the application of narrative analyses here is that the data is generated through participatory and co-creation processes in a group. The narratives that are reported here are co-constructions based on a deliberate participatory process and thus the themes are representative of shared experiences integrated with interpretation to understand the narrative reconstruction. Some of the photos taken and chosen by the members according to the process described in Phase I, are presented below.

## Findings and discussion

Each theme is presented and supported with relevant references and a discussion of theoretical aspects to provide understanding for the members living conditions. In keeping with presentation of narrative analyses, the findings and discussion are integrated. An overarching theme that could be identified in all the members´ experiences in everyday life was that many solutions in society were “*an adaptation for* us, *but it works for no one”*. For clarity and presentation purposes, three aspects of this theme have been used to deepen and report the findings in narrative contexts: “*Accessibility—a never-ending project”, “Tensions of a normative view”*, and “*Power to influence”.*

### An adaptation for us, but it works for no one

The central theme that cuts through the experiences collected in this project, condensed and in the group members’ words, deals with society’s solutions being “*an adaptation for us, but it works for no one”*. This theme has to do with the insufficient integration of first-hand experiences of adults living with SB, in the planning and implementation of social structures intended to support everyday life. Despite solutions such as transportation services enabling community mobility, governmental subsidies to ease employment, and certain adaptations of the physical environment, members in this study highlighted through their examples that many of these systematically developed services and adaptation did not work. Sometimes it seemed that the service design had not included people living with different kinds of disability. For members in this project, this translated to a “struggle in everyday life.” In the following quotation, the predicament is clearly depicted: The landlord had updated the balcony doors in the apartment, which had resulted in a secondary effect of removing the ramp over the threshold to the balcony.

Then he [the landlord] called back and said it was a case for the home adaptation agency, and I, like, how can that be? There are six handicap-adapted apartments in my house in a straight line below me, and everyone has got it like this: no one got help. So, then I wrote to the home adaptation agency, which wrote back that it’s the landlords damn responsibility to redo, to put a ramp back, and I can’t take it anymore.

This description is relevant as it exemplifies the struggle that imbues everyday life for the members of this photovoice project. The Housing Adaptations Grants Act in Sweden (SFS 2018:222) enables people with disabilities to live independently in their own home through housing adaptations grants. According to the act, the municipality is responsible for the housing adaptation. Once this is in place, the landlord needs to ensure that the in-home accessibility is maintained. In this case, it would seem reasonable that when the landlord performed the renovation, he should have restored the accessibility adaptation carried out previously. The above description is an example of how different stakeholders with the responsibility to support individuals with disabilities, pass the responsibility back and forth, placing the individual with disability in a position of uncertainty. In this case financial interests could have been a probable explanation, although they should not have come into play in a seemingly simple and inexpensive restoration of a ramp in accordance with The Housing Adaptations Grants Act in Sweden (SFS 2018:222).

Most of the time, members of the group felt that they did not have the energy to “make a fuss” in stiuations in which they experienced being devalued as a person, whether at work or in a public service situation such as at a restaurant. A clear desire of the participants was to be valued. The issue of checking the box on accessibility yet not offerering good enough accessibility was always present. This impacted on planning of a day, where to go, how to get there, and how to get in. The accessibility of one link in the process could interfere with another, making the ultimate outcome out of reach. Not knowing whether a place was accessible to wheelchair users was linked to worrying about going at all.

At work, employer subsidies when hiring persons with disabilities were raised as an example of an adaptation decided by society, that seems reasonable but in practice has its challenges. To have a job was seen as an advantage in that it meant a place to go, and a context to be part of where one could be active with others in a socially sanctioned way of contributing. For members in this study, going to work was a personally rewarding and meaningful part of everyday life. However, their stories also included descriptions of just sitting around without anything to do for a whole day and not knowing whom to ask and where to go, something in line with other studies (Asaba, Aldrich, Gabrielsson, Ekstam, and Farias, 2020). One of the members brought a photograph of a closed door, representing the inaccessibility to an absent supervisor:

**Figure:**
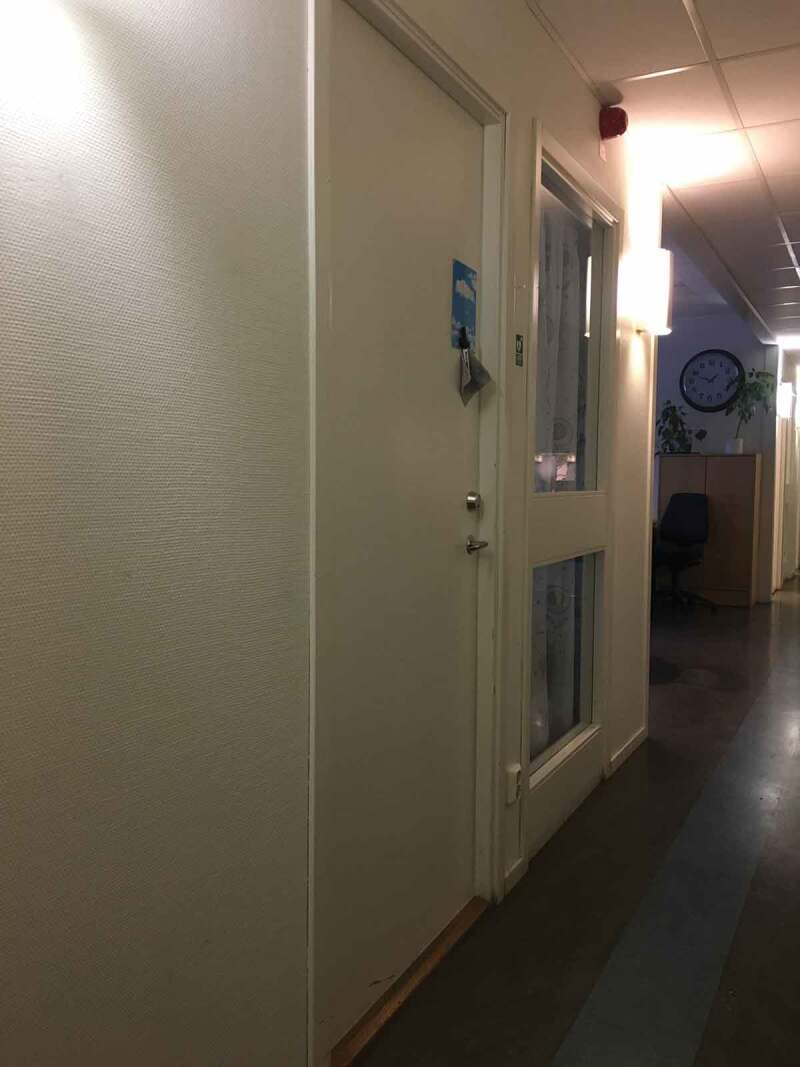
Door in hallway

No one really cares that you should have something to do, something meaningful to spend your time…it looks very much like that at work, that several (persons) go to meetings, sometimes it takes an hour, sometimes it takes half an hour, sometimes it takes several hours. Many who work there often end up sitting for several hours and have nothing to do. So that … I think like, they have, after all, I am employed on something like this contribution and stuff, so it does not cost. They get the contribution to have me there as an OSA [Offentligt Skyddat Arbete (public sheltered work)] employee, or whatever it’s called, so they do not earn, or they do not lose much on having me here.

The members of this project, expressed frustration about feeling like a “token” disabled person in the workplace. This may also happen because employers feel unsure about how to create relevant work situations. The relevance here, however, is that the members’ in this study illustrate how formalized policies and laws, which are in place to ease participation for persons with disability through adaptions or economic support, do not work as intended for some individuals. The result can be social exclusion.

### Accessibility—A never-ending project

Negotiating everyday life included dealing with accessibility. Physical barriers at work, such as a broken elevator, were disenfranchising in several ways. In the following example, after one of the members informed a supervisor about a broken elevator, it took a long time to resolve the matter.

It is good to have a routine sort of, so that you know what to do; but [you may also need to know that] the elevator may be broken, and then you may need to walk through the garage, and when you try to talk to a supervisor they may say that they understand your dilemma but may not do anything about it in a timely manner.

In the scenario described above, the same building also had a health centre, which meant other persons with disabilities frequented the facilities. For the member in this project, the feeling of helplessness about not having an impact on getting the elevator fixed for his own accessibility was as troubling as feeling responsible for the inaccessibility that it caused others in need of visiting the building. The feeling of responsibility was recognized by another participant in the group as well:

X: I do meet older people with wheelchairs or crutches or canes and rollers—I don’t know how many times I had to tell them that I work there. I tell them that I talk to the property manager, but they kind of don’t care about me. I don’t really understand.

Y: Don’t tell them you work there, then you don’t have to take the blame, which isn’t yours.

X: Exactly. It’s a bit frustrating, and at the same time I know that if I quit there because I get pissed about the situation, then I have nothing else to do. No, I don’t know, a supervisor should be interested, whether it is a person in a wheelchair or a person that can walk, that the person can physically access his or her work. Or have the prerequisites to be at work.

Article 9 in the CRPD (United Nations, 2014b) deals with accessibility, which is a necessary condition for people with disabilities to live independently and participate fully and equitably in society. Accessibility should be seen as a disability-specific confirmation of the social aspect of the right of access and availability (United Nations, 2014b). The abovementioned example involves a health centre, which should meet the regulations for a public facility regarding requirements for accessibility in accordance with the CRPD. The member of the study group, who himself used a wheelchair and worked in the building, felt responsible for the inaccessibility towards others in need of an elevator. The member was happy to have a job, which meant somewhere to go during the day, although he was somewhat frustrated as he did not know whether he would actually get into the workplace or, once there, whether meaningful work tasks would be available.

Employing a person with subsidies, as in this case, can in one way be seen as acting in compliance with CRPD Article 27 (United Nations, 2008), by supporting employment for persons with disabilities. Concurrently, other aspects of Article 27 seem to have been neglected by employers, for example, the provision to ensure that reasonable adaptation at the workplace be made for people with disabilities. The CRPD has been said to be based on the human rights principles, guided by values such as inclusion, equality, and human dignity (Degener, 2016). These values have in the abovementioned example not been supported; instead, the message sent was twofold: You have a job but do not really get access to it; and you get to be employed, but without anything proper to do. As a result, the participant was left with no sense of inclusion, and, instead, felt excluded. It is worth mentioning that two of the other members of the photovoice group had sheltered work, which is considered to prevent inclusion in, and interaction with, the community, according the United Nations Committee on the Rights of Persons with Disabilities (United Nations, 2017).

To commute to and from work and to get around in society, most of the participants used transportation services. The question about what to tolerate, regarding transportation, became a topic of discussion for the group. For instance, is it relevant that individuals with disability often have to choose between safety and having transportation. In the picture of a broken seatbelt in the transportation services vehicle, the members wanted to depict a situation that came up too often. The illustration is about constantly negotiate help in securing the seatbelt and double-check it to make sure it was working. If the seatbelt was faulty, one had to consider, whether to prioritize getting to an appointment on time unsafely, or travelling safely but being late.

**Figure:**
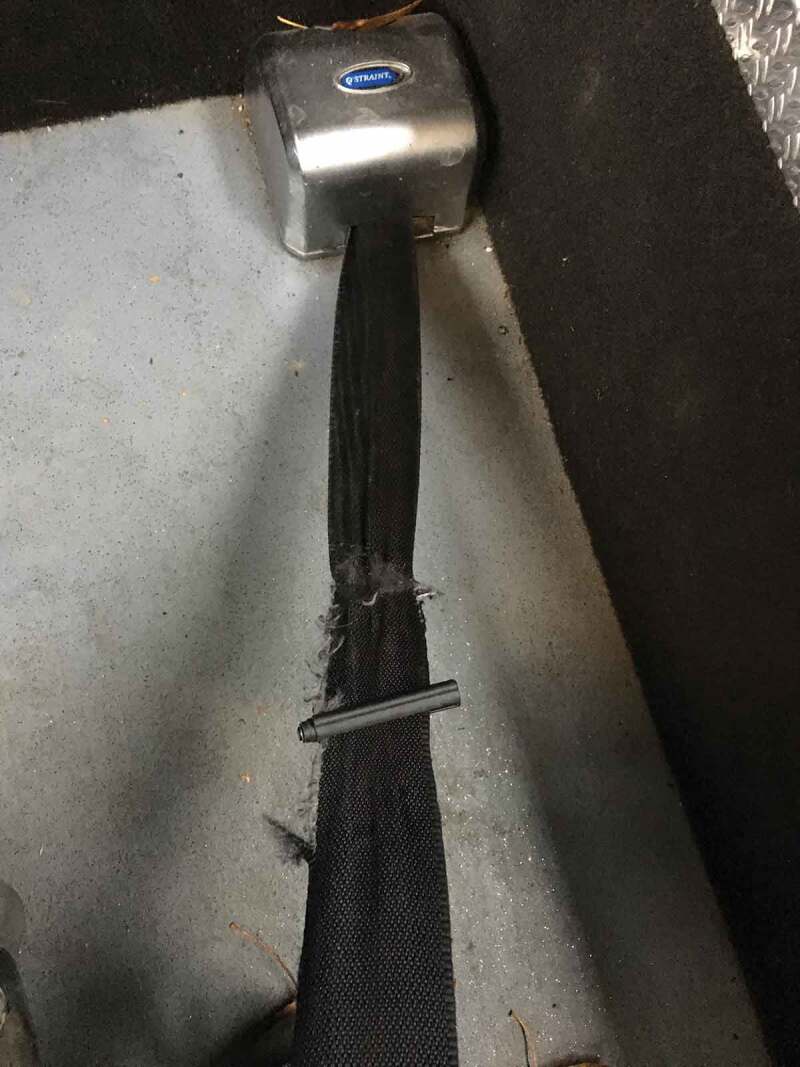
Seatbelt

I happened to have a taxi driver this morning, who was really nice, but he forgot to strap me in. But as a customer I don’t think you should have to remind them (taxi drivers) about that.

Like, if someone suddenly brakes because some other car comes speeding down the road … if a taxi driver is stressed and doesn’t think about this thing about passengers safety and it’s like it’s rushed to go get the next person and things like that, then it’s like safety and those of us who need to ride with this stressed driver like …

Security was also mentioned when the issue of fire drills at work was brought up in the discussion. Not being included when the work place was having a fire drill (when the elevator was not supposed to be used) contributed to feelings of exclusion and unsafety.

### Tensions of a normative view

When the photos from all previous sessions were viewed together, the group noticed that many of the photos depicted a closed door. Photos of closed doors were part of the stories about both physical inaccessibility, and meetings taking place behind closed doors (to the exclusion of the person with disability). Meetings behind closed doors also meant that the supervisor was absent, as previously mentioned. The door had a metaphorical meaning:

**Figure:**
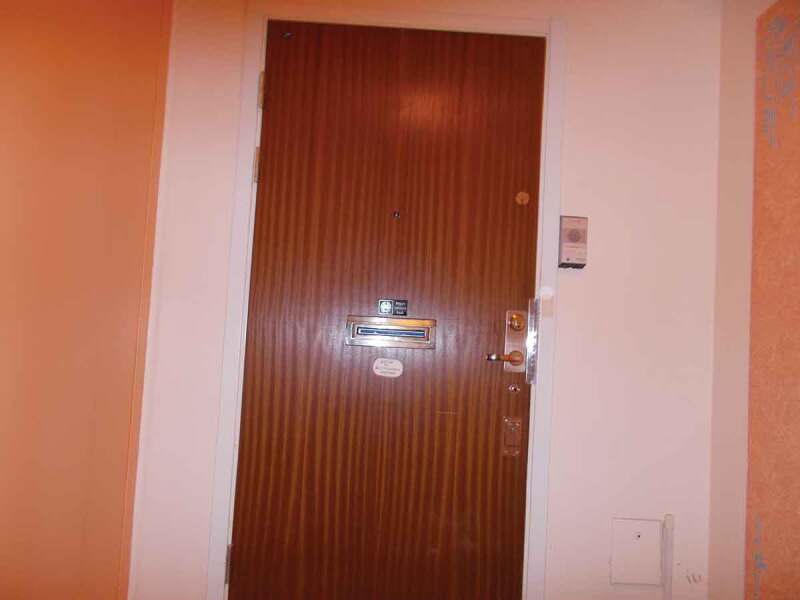
Door to apartment in hallway

There is so much to talk about with the authorities—and things to fix and do—or travel somewhere—or talk to the FK [Social Insurance Office]. And there are many closed doors everywhere. So sometimes you would like to be free as a bird and just, like, [say] No, I don’t give a shit! Now I’m going to leave. No one else should care [about what I do]. I don’t want to book any transport service, I don’t need to use an elevator that’s broken, or I do not—I’ll come out when it’s—oh no, can’t go now, when it is snow in the city

The group did also recognize the potential of a door: as a routing or signpost. However, the issue of physical accessibility, symbolized by a closed door with no possibility to open it, had to do with the way disabled people were treated and, thereby, valued or devalued.

Having grown up with a congenital disability, such as SB, individuals with disability are confronted with normative thinking. The attentiveness of, and response from, the surroundings usually affected the mood of the participants. Cardillo (2010) explored the experience of difference and its intersection with communication for those growing up with chronic illness or disability. She examined autobiographies of persons with disability to obtain insight into possible ways of constructing and making sense of their experience of difference with reference to the communication that surrounded them as they grew up. The narratives showed that communication from others around them partly formed them to become who they were now. Such communication was either directed to disabled persons directly or it could be about other persons with disabilities or simply about disablity in general. This form of communication could be either disconfirming through rejection, dehumanization, and/or abuse, or supportive, respectful, and/or empowering (Cardillo, 2010). The following quotations are examples that the participants gave of a negative attitude in, and degrading comments from, their surroundings:

X: Yes, there was someone at work who said that it would look much nicer if I was sitting in an office chair and I just said, I’m used to sitting in a wheelchair. They really meant that …—it happened to be one of the better supervisors, so—it was a weird comment. I got a bit, I was like annoyed most.

Y: I think it sounds like a comment from an average “normal disordered” person

Z: Yes, exactly.

One member reflects over the fact that the parents wanted him to walk as much as possible. The group member said, “There was something like, You should sit in a wheelchair as little as possible. Yes, it is better the way it is now.” The surroundings communicate, both through providing physical accessibility and through personal communication, sometimes directly with the person and sometimes indirectly, as in the examples above. Constant exposure to this type of normative view from the surrounding public affects the person’s view of him or herself, and of others in a similar situation.

Another, and perhaps more dramatic example, was that of prenatal diagnoses. Prenatal diagnosis are done with the aim to screen for deformity and give pregnant women the option of selective abortion to minimize the risk of having a child born with SB. One of the members in the group reflected that women expecting a child with SB often choose to get an abortion; without placing value in the issue of abortion itself, this group member wanted to assert that individuals with SB should be heard regarding their life experience and also their position on issues such as selective abortion for disability:

… opportunity to influence, if you have SB for example, that you actually get to think something about the fact that there are so many expectant women who get advised to abort fetuses that are expected to get SB. No matter what you think about it—that you actually get to have an opinion about it! That it is not only decided over our heads. … I know some people who have been expecting children who have received very scant information [and have been told] that, Yes, this child, if you choose to give birth, will need a lot of health care, will need a lot of supervision, will cost a lot of money, will not live a full life. It feels like a blow in my face, to be honest … but I AM the one who needs to answer that, that’s how it feels!

What is poignant here is the apparent clarity, not only about the impact of normative communication on identities, but about the paradox lingering between an inclusive society accepting disability and a society that actively discourages the birth of persons that might possible have disability. A common view on selective abortion within disability studies is that a culture that devalues life with disability, devalues people with disability (Ouellette, 2015). There is an underlying ambiguity in the subject, put into words by Asch (Asch, 2003):

Is it possible for the same society to espouse the goals of including people with disabilities as fully equal and participating members and simultaneously promoting the use of embryo selection and selective abortion to prevent the births of those who would live with disabilities? (Asch, 2003, p. 315)

Asch states that revamped clinical practice and social policy could permit informed reproductive choice and respect for current and future people with disabilities (Asch, 2003). Neville-Jan (2005) questions the messages conveyed regarding SB, in the context of preventive measures and selective abortion, saying that they are predominantly negative and that the perspective and lived experiences of persons with impairments should be sought regarding this sensitive subject (Neville-Jan, 2005). This is also evident in Asch (Asch, 2003), who argues that selection against disability through screening and selective abortion is based on, and maintains, misinformation about the lived experiences of children with disabilities and their families (Asch, 2003).

### Power to influence

Power to influence includes opportunities for participation by being asked, in a fair manner, about your own views. This is crucial for access to conditions for inclusion. Participating means being part of society, with focus on autonomy including the ability to act as you wish, and including the opportunity to exert choice and control over how you live and act (Cardol et al., 2002). This should include being asked, in different situations, about your health and your own body, which did not always happen, according to some of the participants. Even when it did happen, the question was asked too late; for example, if it was ok to let a trainee ride on a transportation service bus when the trainee was already on the bus and it was hard to say no. This is also shown in the following quote:

Or, like, when you were little and went to the doctor, or when you were at a treatment home and they would do some surgery—it has actually happened. You come in to a gym, and there sits the doctor who has taken the decision to operate, and then you lay on a mattress and there are 25 medical students sitting next to it. And then no one asks you when you’re 8, 10 years, or so if it’s okay that they sit there. And then when to turn and strap on the legs, and look here and they should squeeze and go on … when talking to them in a single room, after all, yes it might not be 25, but it felt like it. That´s nothing you question when you´re 8,—no, you got to get out.

The above is a memory of an experience from childhood that reflects an exposure and exclusion. It also testifies to an early experience of not being asked, something that had continued the members into adult life. The following quote implies a desire of the participants to be independent, to participate on their own terms, in situations where they can, even when the people around them are trying to be helpful:

Y: You never have to open a door, because there always rushes out some kind old aunt, or some kind of small youth and opens, without me having asked for it. I can do it myself! You never get that far, whether you have a personal assistant or not.

X: That’s it, yeah.

Y: There’s also, perhaps not always, but sometimes, there is a desire for independence. I can do it myself! But you never get there, because someone is already there.

X: But they don’t think about that. I recognize it too. It has happened that people come running from behind and then they just push without saying anything …. I want them to ask.

Not be asked, not be counted, contributes to a sense of social exclusion and not being part of a context characterized by mutual respect. The situation described contains a dimension that concerns how the way your surroundings view you is constantly present.

Participating in an everyday task such as doing laundry was described as an obstacle even though help for handling the task was provided. At times, the help providers did not show up as planned or did not have the time. This made the participants question whether it was better to just solve any problems themselves even if it meant coping with an inaccessible booking system and laundry room.

**Figure:**
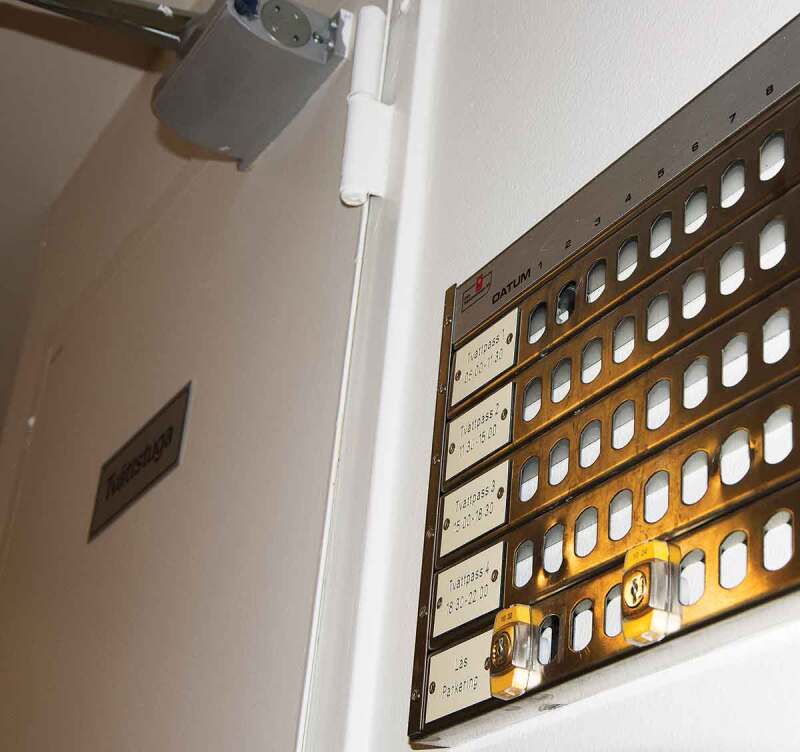
Laundry board

X —I have had a washing machine in the apartment before, so umm no, just as a I started to consider removing that part of home services to, like, do the laundry, like it feels better to actually do laundry myself.

Y —Don’t remove something, add something instead so you get more reimbursement and therefore more rights for personal assistance. Don’t remove something, cause you never know when you’ll need it.

… and then it happened, which was totally not planned, and I don’t recommend. But I sometimes jokingly say, sort of suggest having a seizure. Because when I got seizures, then I got full-time (assistance), 24-hours a day.

The story about this member getting 24-h assistance after having had an epileptic episode can serve as an example where presence of medical diagnoses is often the deciding factor in getting personal assistance. Many adults with SB would benefit from personal assistance. Having assistance can prevent secondary complications; also, it may help in compensating for cognitive impairments. There was an awareness among the group of the ongoing changes in Sweden around the time of the sessions; for instance, the new legislation had overlooked the need for support and service, including personal assistance, among persons with certain types of disabilities. There even had been demonstrations, and two of the participants had attended them. The group discussions often brought up this topic. This shortfall of the legislation happened despite the fact that, in Sweden, not many adults with SB receive assistance allowance, since you have to fit into either of three categories to qualify for it, according to the Act concerning support and service for persons with certain functional impairment (SFS 1993:387).

In many cases, therefore, the help provided from society is not enough, as also shown by a study on the adult SB population in Stockholm, in which, of the persons (44%) who received municipal or state assistance, almost half (48%) relied on additional help from relatives (Bendt et al., 2020). Article 19 in the CRPD deals with the right to live independently and to be included in the community. The UN Committee on the Rights of Persons with Disabilities has identified, in a general comment regarding Article 19 (United Nations, 2017), core elements in the Swedish legislation that need to be adequately addressed, particularly in times of financial or economic crisis. One of these is the right to live independently without having to depend on informal support from family and friends. The UN Committee further stated that addressing these core elements is the responsibility of state parties. Moreover, in a concluding comment to the Swedish government, the UN Committee raised concerns regarding municipal variation in providing personal assistance. They identified a serious gap between the policies followed by the state party and those followed by individual municipalities with respect to the implementation of the Convention (United Nations, 2014a).

## The exhibition

The exhibition opened on the World Spina Bifida Hydrocephalus Day October 25th, 2018, and the exhibition has been touring for 1 year. It has been shown in 10 separate locations, included a library, university college, university and rehabilitation centres in Sweden and Norway. Members in the group have been active in proposing locations for the exhibit and many have been present at the openings. At the very first opening, all the members were present and local politicians and members from patient organizations were invited, and participated in a public conversation where issues from the exhibition were discussed. This kind of event can serve to raise awareness and initiate a dialogue about everyday life with SB, and as a platform for meetings that otherwise not would have come to fruition. This can be viewed as a way to begin contemplations about what social change can be in practice.

## Reflections on possible relevance

The members of this project had valuable experiences of living with SB in Sweden, which comprised having grown up with a congenital disability, in most cases considered as a ´patient´ from birth. This means that the members also had experiences of the health-care system and of treatments within this system, a system that so far does not seem to have asked to hear the views of persons with SB. This may be changing, with more demands on participation within the health care services. Participation in decisions concerning care and treatment has been identified as important in other areas of health care, including that the person can acquire and apply knowledge of symptoms, illness, and treatment (The Swedish Agency for Participation, 2016). Shared decision-making and support for self-care are concepts of relevance in person-centred care where the starting point is that the participation is self-chosen and is based on the person’s conditions and preferences (Ekman et al., 2011). The recent evaluation of methods aimed to improve the conditions for patient participation in health care (focusing on long-term illnesses and conditions), by the Swedish Agency for Health Technology Assessment and Assessment of Social Services (2017), reports some evidence that self-care support efforts provide lasting positive effects on outcome measures related to patient participation. There is a risk that persons who lack, or are not given, the conditions for participation will not receive appropriate care (Swedish Agency for Health Technology Assessment and Assessment of Social Services, 2017). Giving the conditions for participation to persons with SB makes the feasibility of participatory methods used in this study of special interest.

## Methodological considerations

When supporting participatory processes there is much to consider. In this study, efforts were made to follow the will of the group, and of the individuals in the group. The researchers have worked actively with participation throughout the process, through communication methods that develop relations of trust and open communication. These elements are found to relate to increase rigour and trustworthiness (Lennie, 2006). Even though the project was introduced by the researchers, which can be viewed as a limitation concerning the degree to which questions came from an insider perspective, the subsequent steps in the research process followed participatory principles in the choice of themes, photos to discuss as well as photos and captures when creating an exhibition. In this way, the members of the photovoice group had an impact on how the project evolved in terms of which questions were asked. Several members have actively been involved in suggesting locations for the exhibition and been present at the openings. The exhibition and the fact that it has been shown in several locations is considered a strength. However, power relations are always present. In order to levy this gap to the greatest possible degree, members of the photovoice group were included as experts on their situation and as direct insider voices for their experiences. The question of power relations is of importance and can be further developed, as well as the ethical dimension when involving persons considered to be vulnerable and when using photos that may contain persons, for example. The members of the group were asked whether they wished to contribute to the scientific writing; however, none of the members chose to do so.

## Conclusion

This study, through the use of photovoice, describes a hardship in the context of everyday life among persons living with SB. The central theme, *An adaption for us, but it works for no one*, acknowledges this hardship, which is largely based on insufficient integration of the affected individuals’ experience in society’s efforts to plan for, and support, these individuals. The three themes “*Accessibility—a never-ending project”, “Tensions of a normative view”*, and “*Power to influence”* elaborate the central theme, exemplified by broken elevators, unsafe transportations, closed doors, and not getting the opportunity to be involved, all adding to the feeling of not getting the prerequisites to participate. It is of utmost importance that the voice of individuals with SB be taken into account when designing and implementing interventions made to facilitate and/or improve their daily lives.
